# Exoscopic microneurosurgery in pediatric brain tumors: an ideal tool for complex and peculiar anatomo-topographic scenarios?

**DOI:** 10.1007/s00381-023-06138-1

**Published:** 2023-09-11

**Authors:** Andrea Trezza, Camilla de Laurentis, Giorgio Giovanni Carrabba, Maura Massimino, Veronica Biassoni, Arianna Doro, Chiara Vimercati, Carlo Giorgio Giussani

**Affiliations:** 1 Neurosurgery, Fondazione IRCCS San Gerardo dei Tintori, Via Pergolesi 33, 20900 Monza, Italy; 2 School of Medicine and Surgery, University of Milano-Bicocca, Milan, Italy; 3 Pediatrics, Fondazione IRCCS Istituto Nazionale dei Tumori, Milan, Italy; 4 Pediatrics, Fondazione IRCCS San Gerardo dei Tintori, Monza, Italy

**Keywords:** Exoscope, Pediatric neuro-oncology, Micro-neurosurgery, Operating microscope

## Abstract

**Purpose:**

Since its introduction in the 1950s, the microsurgical paradigm has revolutionized neurosurgery. New technologies have been introduced over the years trying to overcome limits of the classical operating microscope. The recently developed 3D exoscopes represent a potential new paradigm for micro-neurosurgery. We analyzed our own experience with a 4 K-3D exoscope in a series of pediatric brain tumors to verify its advantages and limitations in comparison to the operating microscope and in light of the literature.

**Methods:**

Twenty-five pediatric patients with brain tumors underwent surgery at our Institute; the population has been analyzed and described. A score to evaluate the exoscopes and compare it to the operating microscope was considered and postoperatively applied to each single case.

**Results:**

The exoscope appears to be at least comparable to the operating microscope (OM) in all analyzed aspects. In the case of deep-seated or fourth ventricle tumors, the exoscope seems to be superior to the microscope. A surgeon-dependent learning curve is necessary for neurosurgeons to be confident with the exoscope.

**Conclusion:**

Exoscopes appear to be as safe and effective as operating microscopes in pediatric neuro-oncological surgery. They have some advantages that make them superior to microscopes, particularly regarding surgeon ergonomics and fatigue, visual field qualities, and higher choice of intraoperative viewing angles.

## Introduction

Pediatric brain tumors are often considered surgically challenging tumors because they frequently develop in anatomically confined and deep areas such as the posterior fossa, ventricles, and midline structures [1]. Moreover, they tend to reach considerable volumes before causing neurological impairment due to the higher physiological compliance in children leading to an increase in intracranial pressure and the development of focal deficits [2]. Because of these features, surgical treatment can be challenging, particularly because of the limited microsurgical windows and access angles to reach deep lesions and the uncomfortable and lengthy surgical positions required. For example, posterior fossa tumors, which account for more than 40% of pediatric brain tumors [1], may require prone positioning, a park bench or a semi-sitting position [3].

The operating microscope (OM) has long been the gold standard for microsurgical visualization, but its limitations in terms of ergonomics and accessibility of appropriate anatomic surgical angles and corridors have led to the development of new technologies. The introduction of neuroendoscopy brought immediate benefits in terms of surgeon ergonomics, reduction of surgeon fatigue, and visibility of hidden anatomic-topographic angles [4].

However, the use of this technique is limited by the shallow depth of field and the technical constraints imposed by the proximity of the endoscope to the surgical corridor [5, 6].

The recent development of three-dimensional (3D) extracorporeal endoscopes (exoscopes) is a technological innovation that could overcome the limitations of the standard microscope and endoscope while combining their advantages. In particular, the more advanced 3D exoscopes allow improved image quality of the surgical field by eliminating the loss of visual information from the light scattered in the microscope due to refraction and diffraction phenomena used to bend the light toward the eyepieces and provide a longer focal length, a wilder illuminated surgical field, and higher illuminance, thanks to a light-emitting diode and a digital camera [7, 8]. In addition, it is ergonomically advantageous because the surgeon looks forward, as in neuroendoscopic procedures, instead of adjusting his head, neck, and shoulder posture to the position of the eyepieces and the angle of the instrument [7, 9]. Finally, the exoscope offers a relevant extension of the working angle due to the greater freedom of movement.

These main features suggest that exoscope may be an ideal tool for pediatric brain tumor surgery because it allows access to deep-seated structures through narrow corridors that otherwise may require uncomfortable positioning.

To review the efficacy and limitations of exoscopic microneurosurgery for pediatric brain tumors, to determine surgeon satisfaction, and to define any major anatomic-topographic regions or tumor histotypes for the use of this tool, we reviewed our monocentric experience in light of the literature.

## Materials and methods

We reviewed a series of 25 pediatric brain tumors treated at our institution between November 2021 and June 2023 using a 3D 4K exoscope (Olympus Orbeye^®^). The surgeries were all performed by experienced pediatric neurosurgeons with more than 20 years of experience in microsurgical skills. For each patient, we recorded demographic data, including age at surgery, characteristics of the neoplasm and surgery, i.e., tumor location and surgical approach, timing of surgery (from skin incision to closure), extent of resection, and pathology. We also analyzed tumor volumes on preoperative and postoperative post-contrast T1 magnetic resonance imaging (MRI) to confirm the intraoperative extent of resection.

At the end of surgery, the surgeon was asked to complete a questionnaire about his personal perception of intraoperative ergonomics, fatigue, and image quality (especially in terms of depth of field and illumination). He was also asked to rate the possibility of multiple viewing angles in relation to tumor location and surgical approach.

For each question, a score was assigned from 0 to 2, where 0 means that the exoscope has inferior performance, compared with the operating microscope; 1 means that there is no difference between the two devices; and 2 means that the exoscope is clearly superior.

## Results

Twenty-five surgical procedures were performed on 23 pediatric patients diagnosed with pediatric brain tumors at our Institution (Table 1). The mean age at surgery was 11.04 years (3 to 17 years old). The mean surgical time was 4.35 h and was comparable to the mean surgical time for craniotomies performed over the years with an operating microscope in our center. Mean hospital stay was 10.95 days after surgery.

**Table 1:** Further data about our patients’ series

	**Age**	**Tumor site**	**Surgical position**	**Surgical timing (hh:mm)**	**Tumor volume in cm**^**3**^	**Residual volume in cm**^**3**^	**Residual volume %**	**EOR**	**Pathology**	**Preoperative hydrocephalus**	**Hydrocephalus treatment**	**Hospital stay (days)**
**Case #1**	10	Posterior fossa, midline	Prone	07:35	63	0	0	GTR	PA	Yes	Perioperative EVD	11
**Case #2**	5	Right cerebellar hemisphere	Prone	05:34	35.7	0	0	GTR	Medulloblastoma	Yes	ETV	14
**Case #3**	17	Right frontal lobe, midline	Supine	08:17	78.1	0.54	0.70%	NTR	Midline HGG H3 – IDH wildtype	No		8
**Case #4**	13	Right temporal lobe	Lateral	03:03	10.8	0	0	GTR	Cavernous angioma	No		10
**Case #5**	14	IV ventricle	Prone	05:45	32.5	0	0	GTR	Medulloblastoma, Li Fraumeni Syndrome	Yes	ETV	21
**Case #6**	11	Suprasellar diencephalic	Supine	04:31	131.9	33.0	25.02%	PR	Craniopharyngioma	No		9
**Case #7**	11	Right chiasm/optic tract	Supine	04:29	12.3	6.74	54.80%	PR	OPPA	No		10
**Case #8**	5	Posterior fossa, midline	Prone	06:25	106.3	0	0	GTR	PA	Yes	ETV	28
**Case #9**	3	Left frontal lobe	Lateral	02:40	3.07	0	0	GTR	ETMR	No		6
**Case #10**	11	Right cerebellar hemisphere	Prone	04:09	12.3	0	0	GTR	Medulloblastoma	Yes	ETV	19
**Case #11**	16	Suprasellar diencephalic	Supine	04:30	15.4	8.77	56.95%	PR	PA	Yes	Perioperative EVD	10
**Case #12**	11	Suprasellar diencephalic	Supine	03:40	47.9	22.4	46.77%	PR	Craniopharyngioma	No		6
**Case #13**	14	Optic Glioma	Supine	05:35	19.1	10.6	55.50%	PR	OPPA	No		6
**Case #14**	10	Posterior fossa, midline	Prone	07:00	64.8	19.8	30.56%	PR	Glioneuronal tumor	Yes	ETV	26
**Case #15**	15	Right frontal lobe	Supine	04:05	41.1	0.49	1.2%	NTR	HGG	No		8
**Case #16**	9	Suprasellar diencephalic	Supine	03:25	6.88	4.38	64%	PR	Craniopharyngioma	No		14
**Case #17**	3	Left frontal lobe	Lateral	03:10	13.10	0	0	GTR	HGG	No		8
**Case #18**	15	Right frontal lobe	Lateral	04:25	60.40	30.80	51%	ST	HGG	No		8
**Case #19**	17	Right frontal lobe	Supine	02:50	3.46	0	0%	GTR	HGG	No		7
**Case #20**	13	Left frontal lobe	Supine	03:00	1.95	0	0%	GTR	Intracranial Sarcoma	No		7
**Case #21**	11	Posterior fossa, midline	Prone	08:00	27.9	0	0%	GTR	Medulloblastoma	No		8
**Case #22**	10	Left frontal lobe	Supine	02:29	7.78	0	0%	GTR	Glioneuronal tumor	No		6
**Case #23**	6	Posterior fossa, midline	Prone	03:06	1.03	0	0%	GTR	PA	No		6
**Case #24**	15	Right insular lobe	Lateral	04:17	3.62	0	0%	GTR	Glioneuronal tumor	No		10
**Case #25**	16	Left mesencephalic peduncle	Lateral	06:00	10.7	0	0%	GTR	PA	No		11

*EOR* extent of resection, *GTR* gross total resection, *NTR* near total resection, *PR* partial resection, *PA* pilocytic astrocytoma, *HGG* high grade gliomas, *ETMR* embryonal tumor forming multilayer rosettes, *OPPA* optic pathways pilocytic astrocytoma, *EVD* external ventricular drainage, *ETV* endoscopic third ventriculostomy

Eight patients had a posterior fossa tumor: two patients had a lesion in the right cerebellum, and the other six had a midline tumor. Four patients had medulloblastoma confirmed on histopathologic examination, with one patient affected by Li-Fraumeni syndrome; three patients had pilocytic astrocytoma, and one patient had BRAF-mutated ganglioglioma. Surgical positioning was in the prone position, and a right lateralized or bilateral suboccipital craniotomy was performed in all cases. The mean surgical time for posterior fossa procedures was 5.47 h. A gross total resection was achieved in 7 cases. In the patient with ganglioglioma, a partial resection was performed because the tumor infiltrating the dentate nuclei bilaterally to limit the risk of posterior fossa syndrome. Postoperatively, no neurologic deficits occurred, and preoperative symptoms (headache and vomiting in all cases, blurred, and double vision in two cases) had disappeared.

Seventeen surgical procedures were performed for supratentorial tumor: 2 craniopharyngiomas (one patient was operated twice), 2 optic pathway gliomas, 1 diencephalic pilocytic astrocytoma, 1 mesencephalic pilocytic astrocytoma, 1 embryonal tumor forming multilayered rosettes (ETMR), 3 high-grade gliomas (right frontal, bilateral frontal, left frontal, and one patient operated twice for very early recurrence), 1 intracranial fronto-opercular sarcoma, 2 glioneuronal tumors, and 2 cavernous angiomas. The mean surgical time for supratentorial craniotomies was 4.03 h. Cavernous angiomas were included because the surgical approaches, the microsurgical techniques, and the evaluation of the impact of the exoscope on them were similar to those involved in oncologic cases.

Craniopharyngiomas were approached by pterional craniotomy, and one case required second-look surgery to reduce the volume of the cyst before proton-beam therapy. In all cases, partial resection (PR) was performed to reduce the risk of postoperative pituitary and hypothalamic deficits.

The two optic pathway gliomas were approached by pterional craniotomy with partial resection to obtain optic nerve decompression and histological diagnosis. In the case of the diencephalic tumor, a transcallosal approach was chosen because it grew predominantly in the III ventricle. Partial resection with decompression of the foramen of Monro on both sides was achieved. The other cases were hemispheric lesions, all of which were approached by a lesion-centered craniotomy. In all cases, gross-total (GTR) or near-total (NTR) resection (> 90% of preoperative tumor volume) was achieved.

Additional data on tumor volumes, hydrocephalus, and its treatment modalities are summarized in Table 1. No intraoperative or postoperative complications occurred. In all optic gliomas, there was improvement in visual function postoperatively, compared with preoperative findings. Complete regression of preoperative left hemiparesis was observed in the patient with right frontal high-grade glioma. The other case of bilateral frontal high-grade glioma died 4 months after surgery due to extensive disease progression despite oncologic treatments.

The results of the exoscope evaluation by the surgeon are shown in Table 2. Notably, we did not register a score of 0 for any of the analyzed items, and the exoscope was always at least comparable to operating microscope (OM).

**Table 2:** Scoring of Exoscope according to the leading surgeon experience

	**Ergonomics**	**Fatigue**	**Image quality**		**Viewing angles vs tumors’ location**
			**Depth of field**	**Illumination of field**	
**Case #1**	**1**	**1**	**2**	**2**	**2**
**Case #2**	**1**	**1**	**1**	**1**	**1**
**Case #3**	**1**	**1**	**2**	**2**	**2**
**Case #4**	**1**	**2**	**1**	**1**	**1**
**Case #5**	**2**	**2**	**2**	**2**	**2**
**Case #6**	**2**	**2**	**2**	**2**	**2**
**Case #7**	**1**	**1**	**2**	**2**	**2**
**Case #8**	**2**	**2**	**2**	**2**	**2**
**Case #9**	**1**	**1**	**1**	**1**	**1**
**Case #10**	**2**	**2**	**2**	**2**	**2**
**Case #11**	**2**	**2**	**2**	**2**	**2**
**Case #12**	**2**	**2**	**2**	**2**	**2**
**Case #13**	**2**	**2**	**2**	**2**	**2**
**Case #14**	**1**	**1**	**2**	**2**	**2**
**Case #15**	**1**	**1**	**2**	**2**	**2**
**Case #16**	**2**	**2**	**2**	**2**	**2**
**Case #17**	**1**	**1**	**1**	**1**	**1**
**Case #18**	**2**	**2**	**2**	**2**	**2**
**Case #19**	**2**	**1**	**2**	**2**	**1**
**Case #20**	**2**	**2**	**2**	**2**	**2**
**Case #21**	**2**	**2**	**2**	**2**	**2**
**Case #22**	**1**	**1**	**2**	**2**	**2**
**Case #23**	**2**	**2**	**2**	**2**	**2**
**Case #24**	**2**	**2**	**2**	**2**	**2**
**Case #25**	**2**	**2**	**2**	**2**	**2**
**Mean values**	**1.6**	**1.6**	**1.85**	**1.85**	**1.85**

0 = worse than operating microscope (OM), 1 = no differences with OM, 2 = better then OM

Ergonomics, i.e., safety and ease of use, was better than that of OM in 15/25 procedures (60%). These patients all had a deep-seated lesion either supratentorial or infratentorial.

Surgeon fatigue was rated as “operator comfort during surgery” and “surgeon fatigue at the end of surgery.” In 15/25 procedures (60%), the use of the exoscope resulted in less surgeon fatigue, compared with OM. Again, in the majority of cases, a deep-seated tumor was the determining factor for this result.

Depth of field and illumination were superior with the exoscope than with the OM in almost all cases (mean score of 1.85 out of 2), with the exception of more superficial lesions, where the two technologies appear to be similar.

Finally, we considered the possibility of using different viewing angles when approaching the tumor, and we analyzed this concept according to the anatomic location of the tumor. It is evident that for deep-seated tumors and for midline or fourth ventricle lesions of the posterior fossa, the exoscope offers a wider range of viewing angles to approach the tumor resection (mean value = 1.85). There is no difference for more superficial tumors, both supratentorial and cerebellar (score = 1).

## Discussion

Since its introduction in neurosurgery by Professor Yaşargil in the 1950s [10], the OM has been the gold standard for microsurgical visualization to provide the best illumination and magnification conditions for surgery on deep and/or extremely small structures [11, 12]. The stereoscopic view provided by the OM and the evolution of this device, such as the automatic balance and the appropriate focal length (200–400 mm) [6, 11], are the cornerstones for facilitating the surgeon during surgery.

Nevertheless, the OM has some disadvantages that cannot be overcome. First, the advantage of stereoscopic vision could also be a disadvantage. The OM provides a straight line of sight that also is “through the lens” [12], requiring constant adjustment of the instrument’s position to maintain stereoscopic vision and proper illumination of the point of interest. These movements are not only very time-consuming [6] but also require adjustments in the surgeon’s posture, leading to ergonomic problems [6, 13–15]. The same problems related to vision and ergonomics affect the performance of the second surgeon even more [6]. In addition, the OM has a limited range of motion, which, combined with the anatomical tumor location and patient positioning, limits or even prevents access to some areas of the surgical field and forces the surgeon to adopt an ergonomically unfavorable posture during the procedure. This leads to increasing fatigue and possibly long-term worsening of the quality of the surgical gesture.

The introduction of neuroendoscopy seemed to be the solution to ergonomics and the ability to reach more distant regions of interest. This instrument offers more comfort to the surgeon, who can look at a screen in front of his eyes, while the length of the instrument allows easy access to deep structures such as the third ventricle [4]. However, narrow surgical corridors and shallow depth of field soon became a limitation of this technique. Endoscopes are typically long, narrow instruments with small lenses that require extreme proximity to a structure to allow adequate focusing (focal length 3–20 mm). In addition, visualization is limited to a linear line of sight that allows only limited movement [6, 16].

The introduction and initial validation of exoscopic optics in the 2000s and 2010s seemed promising, as exoscopes combine the advantages of the OM and the endoscope while overcoming many limitations of previous optical systems [8, 9]. These features could be particularly useful in such a peculiar field of neurosurgery as pediatric neuro-oncologic surgery [17]. Our initial evaluation of this instrument in this specific and challenging field highlighted several features that could definitely change the paradigm of visualization in the operating room.

The exoscope eliminates the need to look “through the lens,” as it consists of a small articulated arm to which is attached the camera that transmits the image to high-resolution screens. The all-digital imaging system allows for extreme magnification while maintaining a detailed and focused view. In addition, light and magnification are always uniform throughout the surgical field [12]. The introduction of 4K technology, which is a unique feature of the exoscope we use at our Institution (Olympus ORBEYE 3D 4K; Olympus, Tokyo, Japan), provides even a greater depth of field [11, 12, 18–20].

In the literature on exoscopic neurosurgery, several advantages over the OM are frequently mentioned, referring to a more ergonomic and comfortable position for the surgeon, a greater range of motion, and consequently a better and larger field of view with improved illumination, image quality and visualization, and magnification of structures [11, 21]. These advantages may be particularly applicable to pediatric deep-seated and posterior fossa tumors, which often force positioning of the patient and require wide illumination, greater depth of field and longer surgical times, similar to complex procedures such as those requiring retrosigmoid approaches [22]. Our experience is absolutely consistent with the findings in the literature (Tables 1 and 2).

When analyzing the data listed in Table 2, the exoscope was consistently rated superior to the gold standard OM in terms of image quality in the majority of our evaluations (mean score of 1.8 out of 2). The evaluation took into account depth of field, intraoperative image resolution, and illumination of the surgical scenario at all levels up to the maximum possible magnification. For more superficial lesions, we found no significant difference between exoscope and OM.

A peculiarity of pediatric neurosurgery is a vivid pulsatility of the brain, which is much more evident than in adults. A potential limitation of the exoscope is the relative magnification of this pulsatility, which creates a great visual effort for the surgeon. This is particularly evident in lobar supratentorial tumors, whereas it is negligible in deep-seated and fourth ventricle lesions. Another factor that could negatively impact the quality of surgical exoscopic vision is the increased vascularization of some tumors, which can lead to continuous intraoperative bleeding and a less defined field of view [12].

Ergonomics and musculoskeletal discomfort are underdiscussed problems in neurosurgery and in surgery in general. Pediatric neuro-oncologic surgery, with its peculiar pathologies and anatomic locations, certainly places greater demands on the surgeon in terms of intraoperative stress and fatigue. Especially in long cases these factors may also have a negative impact on the quality of surgical performance, which is particularly important in a field where surgery is still of primary importance in influencing the prognosis of children [14, 15, 21, 23].

**Fig. 1:**
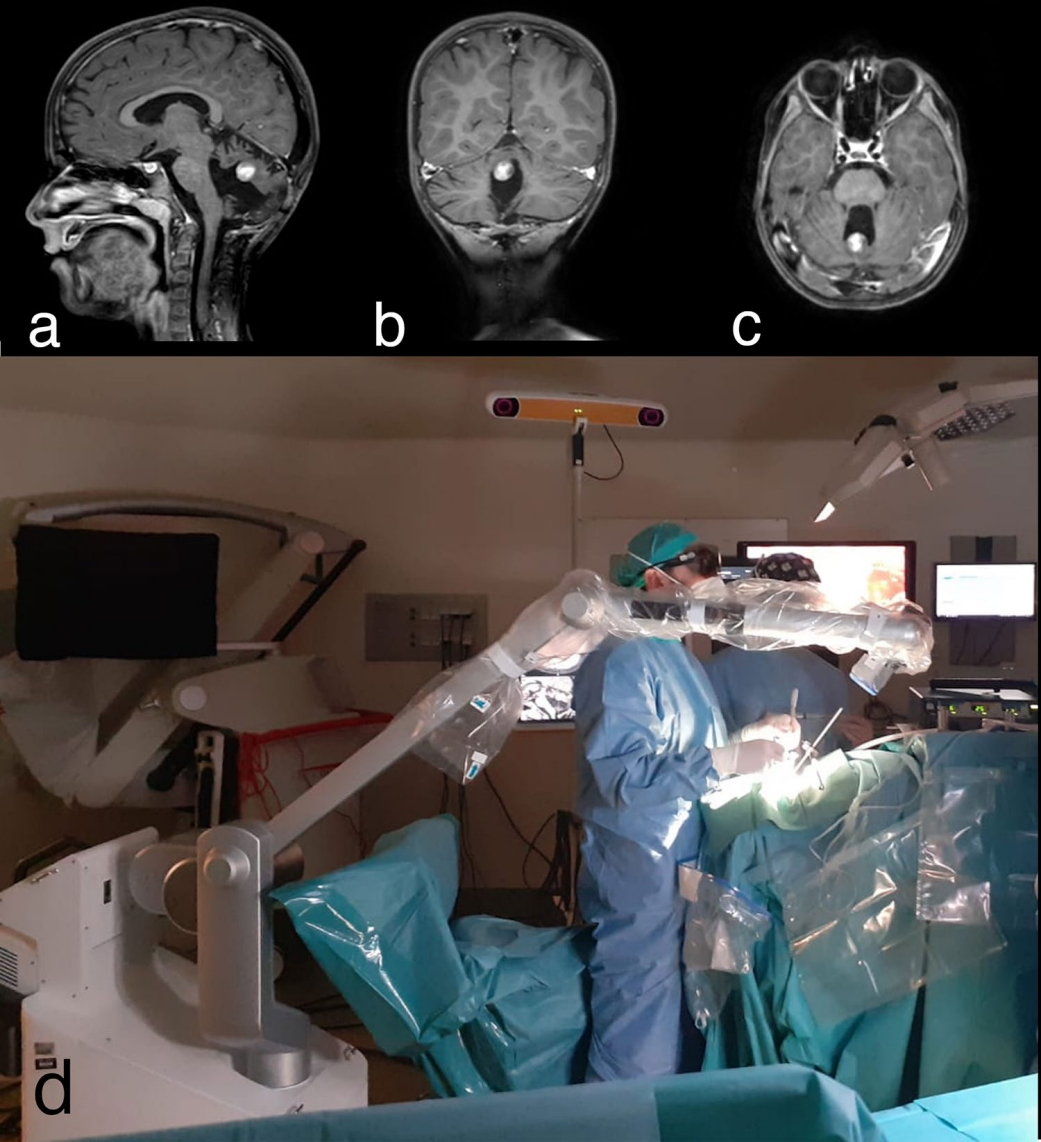
Preoperative T1 post-contrast magnetic resonance images of a recurrent pilocytic astrocytoma in the upper vermis (**a** in sagittal plane, **b** in coronal plane, and **c** in axial plane) and position of leading surgeon and exoscope in the operating room (**d**)

Allowing a more physiologic neck and back posture and dramatically reducing the need to maintain eye contact and a fixed posture for extended periods of time would greatly reduce surgeon fatigue while increasing the safety of surgery [18]. In our experience, the exoscope was slightly superior to the OM in terms of ergonomics and fatigue. Case-by-case analysis reveals that impact on surgeons’ posture is influenced by the location of the tumor, with the exoscope being better in the prone position and for deep-seated tumors than OM (Fig. 1).

As pediatric neuro-oncology often involves deep-seated or posterior fossa tumors, the ability to increase range and freedom of motion without sacrificing image quality and illumination of the field is of paramount importance to the surgeon and can be a determining factor in pursuing surgical radicality, which is still considered one of the most important prognostic factors [24–26]. Consequently, this has a positive impact on the ergonomics and fatigue of the leading surgeon and the entire team, even if it requires a learning curve. In our own experience, the exoscope still seems superior to the OM in this particular anatomic scenario.

The paradigm shift from the “through-the-lens” view of the OM to the direct view of external high-resolution 3D screens requires time for the surgeon to adapt to the new way of working and a consequent learning curve, which may be a potential limitation in the use of the exoscope [18, 21]. On the other hand, the bimanual surgical technique does not change between OM and exoscope, and the continuous and extensive use of the exoscope over a longer period of time would shorten the learning curve and lead to even better performance [6, 20, 27].

As a new surgical paradigm, the exoscope requires a complete recalibration of how surgery is performed. Not only the leading surgeon, but the entire surgical team (especially the second surgeon and the scrub nurse) would have to redesign their intraoperative activities. It is reported that a not insignificant percentage of surgeons have difficulty assisting with the exoscope and that a learning curve is required for the second surgeon to be functional in performing the surgery. Operating room nurses also reported less comfortable positioning during surgery [28, 29].

A generational difference is also evident in older neurosurgeons who have spent their entire careers performing surgery and refining their techniques at the OM. New generations of surgeons may have the advantage of already being accustomed to moving their hands and performing tasks while looking at a screen, such as while playing videogames [30]. They also have the option of using the exoscope from the beginning of their surgical experience and microsurgical learning curve. This would certainly make them more prone to the use of exoscope than OM, shortening their learning curve.

The exoscope also offers an advantage in terms of training, as the screen and surgical view of the leading surgeon are shared equally with the entire operating room (OR) staff, allowing for better participation in the procedure. This would be beneficial for residents and medical students [31] to gain a better understanding of anatomical and pathological issues and micro-neurosurgical techniques. This is of particular importance in a field such as pediatric neurosurgery, where there are many rare diseases and high standards of care are required.

## Conclusion

The exoscope appears to be as safe as the OM in performing micro-neurosurgical procedures for pediatric brain tumors, with even greater advantages for deep-seated and posterior fossa tumors. It allows for better visualization and more comfortable management of the deep portion of the neoplasm while improving the ergonomics and quality of the procedure and reducing surgeon fatigue. As with any medical technology, a learning curve is required, but it appears to be short and feasible, at least for experienced neurosurgeons. Because pediatric brain tumors are a rare pathology and treatment of affected children requires a higher level of surgical performances, the training benefits of the exoscope appear also to be paramount.

## Data Availability

We the authors commit ourselves to provide all data upon request.
